# Cross-Linking and Evaluation of the Thermo-Mechanical Behavior of Epoxy Based Poly(ionic Liquid) Thermosets

**DOI:** 10.3390/polym13223914

**Published:** 2021-11-12

**Authors:** Florian Wanghofer, Archim Wolfberger, Markus Wolfahrt, Sandra Schlögl

**Affiliations:** Polymer Competence Center Leoben GmbH, Roseggerstraße 12, 8700 Leoben, Austria; florian.wanghofer@pccl.at (F.W.); markus.wolfahrt@pccl.at (M.W.); sandra.schloegl@pccl.at (S.S.)

**Keywords:** ionic liquid, epoxy, ionene, poly(ionic liquid), thermoset

## Abstract

Poly(ionic liquids) (PILs) and ionenes are polymers containing ionic groups in their repeating units. The unique properties of these polymers render them as interesting candidates for a variety of applications, such as gas separation membranes and polyelectrolytes. Due to the vast number of possible structures, numerous synthesis protocols to produce monomers with different functional groups for task-specific PILs are reported in literature. A difunctional epoxy-IL resin was synthesized and cured with multifunctional amine and anhydride hardeners and the thermal and thermomechanical properties of the networks were assessed via differential scanning calorimetry and dynamic mechanical analysis. By the selection of suitable hardeners, the glass transition onset temperature (*T*_g,onset_) of the resulting networks was varied between 18 °C and 99 °C. Copolymerization of epoxy-IL with diglycidyl ether of bisphenol A (DGEBA) led to a further increase of the *T*_g,onset_. The results demonstrate the potential of epoxy chemistry for tailorable PIL networks, where the hardener takes the place of the ligands without requiring an additional synthesis step and can be chosen from a broad range of commercially available compounds.

## 1. Introduction

Ionic liquids (ILs), which are generally defined as salts with melting points below 100 °C, have attracted increased attention in recent years in research and industry [1,2,3]. They consist of a bulky organic cation and an organic or inorganic anion [4,5], decreasing the packing density of the molecules and reducing the melting temperature (*T*_m_) compared to conventional inorganic salts [6,7]. Asymmetrical cations and weaker interactions between the ions usually shift the transition temperatures to lower values [8], in many cases even well below room temperature (RT) [9].

The unique properties of ILs, such as low flammability, negligible vapor pressure, moisture resistance, high thermal stability [10], reusability [11], ion conductivity [12], as well as catalytic activity [13,14,15], combined with their high tunability and wide range of available ILs with a vast number of possible anion-cation combinations give rise to many possible applications [10]. Examples are their use as solvents [16], latent curing agents [17], phase transfer catalysts [18], dispersing agents [19], and porogens [20,21], as well as their application in materials for CO_2_ capture [22], magneto-rheological fluids [23], and self-assembly of polymers [24]. Other reports include the formation of ionogels produced from ionic liquids and a suitable gelator, exhibiting high ionic conductivity and tunable properties that can be used as quasi-solid electrolytes [25,26].

**Figure 1:**
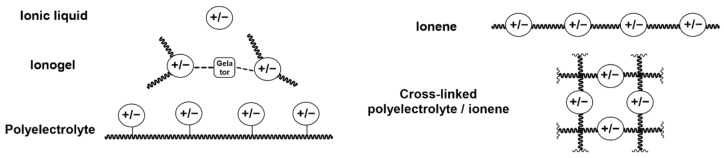
Schematic representation of different poly(ionic liquid) (PIL) structures and terminology thereof.

A further advantageous property of ILs is that they can also be functionalized with a variety of different reactive groups to act as non-volatile monomers for the realization of poly(ionic liquids) (PILs). Depending on their structure and position of the ionic moieties in the polymer chains, different terms are used to describe the resulting materials. An overview of the chemical structure of different poly(ionic liquids) is provided in Figure 1. The properties of the resulting PILs are greatly influenced by their respective ion pairs. Differences in the glass transition temperature (*T*_g_) of 50 °C or more were reported for PILs by exchanging the respective counter-ions of the poly(ionic liquids) [27,28]. Thus, the properties of soluble PILs can be further tuned by anion exchange, even after polymerization [28,29,30]. Exchanging the anion can also be utilized to adjust the solubility of the polymer [31,32] and the mechanical properties [33]. Depending on the monomer structure and the ion pair, PILs can be designed to be hydrophobic [34,35], antibacterial [36], and non-flammable [37] polymers. While the anion selection offers many opportunities to create task specific materials, some anions result in a significant increase in brittleness and the PILs cannot be processed as a consequence [38,39,40].

In many cases, PILs exhibit high CO_2_ solubility and solubility selectivity [41,42], especially in combination with free ionic liquids as solid-liquid composites [42,43,44]. This renders them very interesting candidates for gas separation membranes. The reported loadings of free ILs in the PIL membranes are often above 40 wt.% [45,46], whereas the PIL matrix increases the maximum filler capacity by intermolecular bonding and prevents leakage [28]. Moreover, PILs have shown to be potential candidates as catalysts for solvent free reactions [47,48]. Another interesting property of PILs is their ionic conductivity for applications as polyelectrolytes [49].

Several different approaches to polymerizing ILs have been reported in literature: (i) As pendant group of a polymerizable monomer [50,51], (ii) block copolymers of IL monomers with non-ionic monomers [52,53], (iii) by protonation of amine functional groups and subsequent cross-linking with an epoxy resin [54], (iv) or by using cross-linkable ionic liquid monomers [46]. While polymerizable cations are more common, it is also possible to functionalize the anion or even both ions [55,56]. The majority of the reported PILs are obtained from monomers with only one polymerizable group with the ionic moieties as pendant groups [57,58,59]. By polymerizing these monomers, a polyelectrolyte is formed.

Similar structures can be produced by grafting an ionic group to the backbone as a side chain [57,60,61,62] or via polymerization of a dihalide with bisimidazole molecules [63,64,65,66]. The latter results in ionenes, which are polymers with ionic moieties in their backbone [67]. PILs and ionenes are generally very similar and share the same field of applications [68]. However, while PILs are often based on mono-functional alkenyl moieties, and therefore, possess an aliphatic, non-ionic backbone, literature reports on ionenes increasingly focus on the introduction of ionic groups in high performance polymers such as polyimides [63,69] or aromatic polyamides [64,70]. Furthermore, the position of the ions influences the ion aggregation [71] and an increased ion conductivity was found for ionenes in comparison to a polyelectrolyte [72].

IL-based ionenes can also be obtained from multifunctional ILs [73]. Difunctional ILs equipped with epoxy-functional groups (epoxy-IL) represent a very versatile class of materials. The oxirane ring exhibits reactivity towards a multitude of species and can be used for polymerization with amines, phenols, thiols, isocyanates or carboxylic acids following a step-growth mechanism. However, epoxies can also be polymerized by a chain-growth reaction mechanism, either as homopolymerisation or as alternating copolymerization with anhydrides [74,75]. Epoxy based polymers with thermoplastic properties can be produced by the homopolymerisation of monofunctional epoxy resins or by curing a difunctional epoxy resin with a difunctional hardener [76]. If the epoxy resin or the hardener have a functionality of more than two, cross-linked PILs/ionenes are acquired.

Most reported epoxy functional ILs are based on imidazolium cations, due to their high thermal stability [77], combined with bis(trifluoromethane)sulfonimide (Tf_2_N^−^) anions, which ensure that even the large aromatic monomer molecules are liquid at lower temperatures [78], and yield hydrophobic polymers [34,36,79]. In contrast, epoxies based on the widely used diglycidyl ether of bisphenol A (DGEBA) tend to be more hydrophilic [34,80]. The starting compounds are usually functionalized using halides or tosylates, either by direct epoxidation with epichlorohydrin/epibromohydrin [81,82,83,84,85] or by addition of an alkenyl group and subsequent oxidation of the double bond [42,46,49,78].

Table 1 summarizes literature examples of epoxy-IL monomers and their respective glass transition temperature after polymerization. McDanel et al. [42,46] synthesized PILs 1A–D for CO_2_ capture. The networks were produced by curing the respective monomers with tri- or hexa-functional amine hardeners. Through variation of the hardeners and their stoichiometry, the authors were able to significantly influence the CO_2_ uptake of the materials as well as the respective glass transition temperatures.

Radchenko et al. [34] synthesized the aromatic PIL 2 that can be used for shape memory applications due to its two relaxation temperatures at 60 °C and 109 °C. The authors reported comparably high mechanical properties, with a Young’s modulus of 1.63 GPa, a yield strength of 96 MPa, 28 % strain at break as well as a storage modulus of 1.7 GPa at room temperature. In another publication, the authors produced the three monomer materials 3A–C, which have a high potential for 3D-printing applications in combination with diaryliodonium hexafluoroantimonate as the UV or thermally activated initiator. The cationically cured monomer 3B led to a polymeric network with a storage modulus of 1.18 GPa [86].

Livi et al. [36,49] produced PILs from monomers 4A–C for antibacterial coatings or surfaces and reported a biofilm inhibition of more than 80 % for all three polymers. The PILs exhibited rather high glass transition temperatures between 53 °C and 62 °C, considering that an aliphatic hardener was used. The highest value was reported for 4B, which does not contain an ether bond in the epoxy monomer. The storage moduli are similar for all three polymers, with 700 MPa reported for 4A at room temperature [36,49]. Apart from a reduced stiffness of PILs compared to commercially available epoxy monomers, the ionic character is expected to have an influence on the reactivity of functional groups. While the peak of the exotherm of 4A during curing is observed at lower temperatures than that of DGEBA [49], it is less reactive than monomer 4C with its two aromatic ligands. The authors attribute this lower reactivity to the higher distance of the oxirane ring to the cation [36]. The substituents do not only influence the properties of the monomer but also the synthesis. Electron donating groups on aromatic rings reduce the reaction speed during the oxidation of the alkenyl group to yield epoxide rings. The highest effect was observed for ester groups in the synthesis of various monomers [78].

Grugel and co-workers [79] investigated the properties of epoxy-IL resins in detail to examine their suitability as matrix materials in carbon fiber reinforced composites for the storage of liquid hydrogen and for cryogenic applications. The thermoset resulting from curing of monomer 5 is reported to exhibit low flammability, hydrogen permeability and thermal expansion, as well as increased adhesion, lap shear and tensile strength. Increased impact resistance and fracture toughness were observed, especially under cryogenic conditions in comparison to a commercially available DGEBA based epoxy resin. The reported tensile strength of approximately 60 MPa is significantly lower than for PIL 2 but still within the range of technical resins [79]. It has to be noted that the IL was cross-linked with an aromatic amine hardener, while the reference DGEBA resin was cured with polyoxypropylenetriamine, an aliphatic polyether. PIL 5 was exposed to aging tests in space, facing earth for approx. 2 years, in which it went through 12,500 cycles between −40 °C to 40 °C under the influence of radiation and oxygen. The investigated specimen on aluminum substrates exhibited no cracks, delamination or loss of adhesion after the tests [87]. Furthermore, the material was tested with fiber reinforcements and exhibited increased strain under liquid nitrogen conditions and better adhesion to the fibers compared to a common DGEBA based thermoset. A different crack growth behavior was observed for DGEBA based thermosets and PIL 5. The DGEBA based material exhibited failure along the interface between fiber and epoxy, while the crack propagated through the epoxy matrix for PIL 5 and even plastic deformation before failure was reported [88]. In contrast, PIL 5 did not show any cracks or delamination from temperature cycles between cryogenic and room temperature, and surpassed commercial systems in terms of tensile and bond strength [89].

Another possibility to synthesize IL based epoxy networks is to functionalize the cation with a group that can react with epoxy monomers, such as amines or carboxyl groups. Amine functional ILs can also be obtained by quaternization of an amine curing agent [54], by exchanging the anion to an amino acid, which allows the synthesis of ILs with both functional anions and cations [22,90]. Another route involves the Radziszewski imidazole synthesis using functionalized amines, which is reported for the production of both amine and carboxylic acid functional ILs [91,92].

Jiang et al. [93] synthesized diamine imidazolium hardeners with dicyanamide and Tf_2_N^−^ anions and used it to cure a trifunctional epoxy resin. The authors determined the ideal amount of hardener to be 15 wt.% of IL, which is non-stoichiometric. Therefore, a significant number of cross-links can be expected to be based on homopolymerisation, which is likely the reason for the higher glass transition temperature (212 °C/196 °C) of the network compared to other reported PILs. Apart from a high *T*_g_, the thermoset exhibited good mechanical properties with a tensile strength of up to 40 MPa and a strong influence of the anion. Using Tf_2_N^−^ as counterion resulted in a lower cross-link density and tensile modulus but increased tensile strength and elongation at break. Interestingly, despite the decreased cross-link density, a higher *T*_g_ were reported for the Tf_2_N^−^ anion than for the dicyanamide.

Jin et al. [60] reported a seven-fold increase of impact strength upon the addition of a carboxyl functional polyether with pending imidazolium IL groups to a commercial epoxy resin. It has to be noted that the addition of the polyether also introduces long alkyl chains into the network, which might play a significant role in the impact strength enhancement.

Apart from epoxy groups, amines can also react with anhydrides to form polyimides. Li, Zhang et al. [73,94,95] produced random and block-copolyimides with mono- and di-cationic amine ILs. The addition of different ratios of the amine IL led to an increase in density while it significantly decreased the molecular weight and the glass transition temperature from initial 296 °C to as low as 177 °C for 30 mol% of IL.

Due to this high tunability and sheer endless combinations, numerous reports on the synthesis and characterization of task-specific PILs are available in literature. This, in turn, requires custom syntheses for most PILs. Livi and co-workers simulated the properties of homopolymerized epoxy PILs based on the monomer structure to reduce the amount of different syntheses required [96]. In the present work, the versatility and considerable potential of epoxy-functional ILs is further demonstrated via the selection and implementation of different hardeners, resulting in an effective tailoring of the properties of the prepared epoxy-IL networks. Specific focus is laid on a systematic evaluation of the thermal and thermo-mechanical properties of representative epoxy-IL networks, cured with different types of hardeners. For this purpose, an epoxy-functional IL monomer with comparably short side-chains was synthesized and cured with selected multifunctional amine hardeners. The thermal and thermo-mechanical properties of the resulting epoxy-IL networks are examined and compared to a reference DGEBA based epoxy resin as a benchmark. To the best of the authors’ knowledge, this has not been extensively done so far. The potential for a flexible tailoring of the network properties of epoxy-IL resins is further demonstrated by an investigation of resin systems prepared with varying mixing ratios between epoxy-functional IL and DGEBA as a commonly used benchmark resin.

## 2. Materials and Methods

### 2.1. Chemicals and Materials

1*H*-imidazole, 3-chloroperoxybenzoic acid (77%), 3-(aminomethyl)-3,5,5-trimethylcyclohexanamine (IPD), 4,4’-diaminodiphenylsulfone (DDS), and trimethyl-1,6-hexanediamine (TMHMDA) were obtained from Sigma-Aldrich (St. Louis, MO, USA). 4-bromo-1-butene and lithium bis(trifluoromethane)sulfonimide were purchased from ABCR (Karlsruhe, Germany). 4-methyl-hexahydro-phthalic anhydride (MHHPA) and bisphenol A diglycidyl ether (DGEBA) were supplied by Huntsman (The Woodlands, TX, USA). *N*,*N*-dimethylbenzylamine (BDMA) was purchased from Fisher Scientific (Hampton, NH, USA). All chemicals were used without further purification.

### 2.2. Synthesis and Thermal Curing of Ionic Liquids

1,3-bis(2-oxiranyl-ethyl)imidazolium Tf_2_N^−^ (IL-E) was synthesized according to a procedure published by McDanel et al. [46]. 20.07 g Imidazole (294 mmol) was dissolved in 170 mL acetonitrile and 24.8 g NaHCO_3_ (294 mmol) and 83.57 g 4-bromo-1-butene (617 mmol) were added to the solution and refluxed for 15 h. The reaction mixture was allowed to cool to room temperature, subsequently filtered and the solvent was removed under reduced pressure. 175 mL of de-ionized water were added to the residue, followed by washing with ethyl acetate and heptane. An amount of 88.83 g (309 mmol) of lithium bis(trifluoromethane)sulfonimide was added to the aqueous phase and the solution was stirred for 4 h at room temperature. The aqueous phase was separated, and the remaining oily layer was extracted with 500 mL ethyl acetate. The organic phase was washed with 1 M aqueous HCl solution and saturated NaHCO_3_ solution, followed by washes with de-ionized water until the test with AgNO_3_ was negative for halides. MgSO_4_ and activated charcoal was afterwards added to the organic phase. After stirring for 3 h, the solution was filtered through basic alumina and the solvent was removed under reduced pressure to yield 122 g (90% yield) of 1,3-di(3-butenyl)imidazolium Tf_2_N. The epoxidation was performed batchwise: 45 g (98 mmol) 1,3-di(3-butenyl)imidazolium Tf2N was dissolved in 200 mL acetonitrile, 90 g (394 mmol) 3-chloroperoxybenzoic acid was added and the solution was then stirred for 48 h at room temperature. The reaction mixture was filtered, and the solvent was removed under reduced pressure. Residual 3-chlorobenzoic acid and unreacted 3-chloroperoxybenzoic acid was removed by washing with 2-methoxy-2-methylpropan. The product was dried under vacuum to yield 35 g (73%) of IL-E. The chemical structure was confirmed via ^1^H- and ^13^C-NMR in DMSO-d6, and FTIR spectroscopy using literature reports [46,97,98] (Figures S1–S3, Supplementary Materials).

IL-E was cured with selected amine and anhydride hardeners in stoichiometric ratios. TMHMDA, IPD, and DDS were used in a molar ratio of 0.5 to the epoxy resin. MHHPA was added in a molar ratio of 1.7 to IL-E, with 3 wt.% BDMA in relation to the epoxy resin as accelerator. DGEBA was cured with DDS in a molar ratio of 1:0.5 as a reference thermoset. For the production of resin containing both IL-E and DGEBA, the resins were mixed in the desired molar ratios prior to the addition of DDS in a stoichiometric ratio. Liquid hardeners were thoroughly mixed into the resin at room temperature. DDS was dissolved in the epoxy monomers by stirring the components at 150 °C until a homogenous mixture was achieved (between 5 and 20 min). The formulations were then homogenized in a vortex mixer and degassed under vacuum before curing. An overview of the chemical structure of the implemented resins and hardeners, their respective abbreviation and cure conditions of the networks is listed in Table 2. 

### 2.3. Characterization Methods

Infrared spectra were recorded on a Vertex 70 spectrometer (Bruker, Billerica, MA, USA) in attenuated total reflectance (ATR) mode with the software OPUS 7.5 (Bruker, Billerica, MA, USA). A total number of 16 scans were gathered from 500 cm^−1^ to 4500 cm^−1^ with a resolution of 2 cm^−1^.

Measurements of the viscosity of the investigated resins were done using an MCR 501 rheometer (Anton Paar, Graz, Austria). The plate-plate measurements were conducted with a shear rate of 10 s^−1^ and a deformation of 7% at 30 °C.

A dynamic mechanical analysis (DMA) was done on a DMA/SDTA861^e^ instrument (Mettler-Toledo, Columbus, OH, USA) in tensile mode with a clamping length of 9 mm in accordance to DIN EN ISO 6721-4 [99]. Rectangular specimens of 20 mm × 2.5 mm × 1 mm were tested in a temperature range of 50 °C up to a maximum temperature of 250 °C or at least 100 °C above the maximum tan delta with a heating rate of 2 K/min under nitrogen. All measurements were performed in the strain-controlled mode with 3 µm deformation, 1 Hz frequency and 150 % off-set.

The thermal analysis equipment DSC 4000 (PerkinElmer, Waltham, MA, USA) was used for differential scanning calorimetry (DSC) measurements according to DIN EN ISO 11357-2 [100]. The weight of the samples in the standard aluminium pans (40 µL) was in the range of 12 mg to 14 mg for cured samples and 4 mg to 6 mg for uncured samples. All samples were first heated from −50 °C to 200 °C or 300 °C, for resins containing DDS, with 20 K/min under nitrogen atmosphere. Afterwards, each sample was cooled down to −50 °C. In the subsequent second run, all samples were heated up again to the above-mentioned temperatures using the same heating rate and atmosphere. The glass transition temperature was determined as midpoint temperature in accordance to the relevant standard using the software Pyris (PerkinElmer, Waltham, MA, USA).

^1^H-NMR and ^13^C-NMR spectra were recorded on a Bruker (Billerica, MA, USA) Advance III 300 MHz NMR spectrometer in DMSO-*d6*.

Thermogravimetric analysis (TGA) was performed on a TGA/DSC thermogravimetric analyser (Mettler-Toledo, Columbus, OH, USA) from 25 °C to 900 °C with a heating rate of 10 K/min under nitrogen in aluminium oxide crucibles with a volume of 70 µl and an approximate sample weight of 30 mg. The measured curves were evaluated with the STAR^e^ Evaluation Software (Mettler-Toledo, Columbus, OH, USA)

Density was measured in distilled water using a 50 mL pycnometer with a ground-in thermometer at room temperature. The density of the samples *ρ_s_* was calculated from the sample weight *m_s_*, the weight of the pycnometer filled with water *m_w_*, the weight of the pycnometer filled with water and the sample *m_sw_*, and the density of water at the respective temperature *ρ_w_* using the following equation:

$$\rho_{s}=\frac{m_{s}}{m_{s}+m_{w}-m_{sw}}*\rho_{w}$$

## 3. Results and Discussion

The synthesis of the ionic liquid 1,3-bis(2-oxiranyl-ethyl)imidazolium trifluoromethanesulfonimide (IL-E) via alkenylation followed by ion exchange and epoxidation [46] proved to be a very robust and efficient procedure for the production of epoxy-ILs with high purity, as also indicated by the obtained NMR spectra and the nearly colorless appearance of the IL [101]. The monomer was chosen for this study due to its chemical structure with comparably short side chains. This allows for more versatility, since the network properties can be effectively tailored and repeating units between the ionic moieties can be easily varied by the hardener. IL-E exhibits a viscosity of 150 mPa s at 30 °C and was stable during storage for several months. In comparison, a significantly higher viscosity of 860 mPa s was measured for the commonly used epoxy resin DGEBA.

### 3.1. Thermal Properties of Thermally Cured IL-E

**Figure 2:**
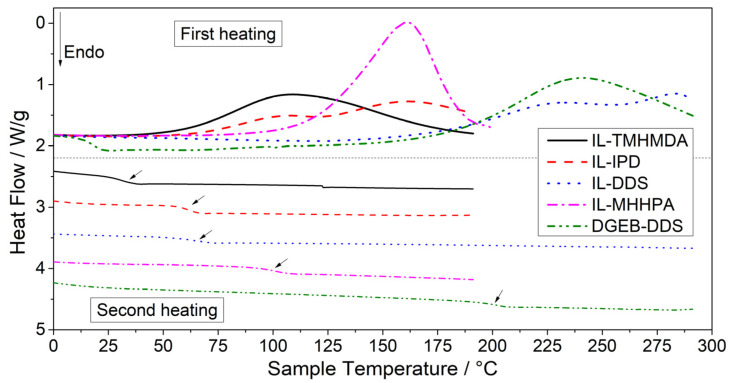
DSC thermograms of the uncured resin-hardener mixtures, including the first (top) and second (bottom) heating. Curves shifted vertically for better visibility.

The epoxy-IL resins were thermally cured with aliphatic, cycloaliphatic and aromatic amine hardeners to achieve cross-linked networks with different chemical and physical properties. Their curing behavior was monitored via DSC and complete curing was characterized during the second heating run. Due to the higher reactivity of the systems including aliphatic and cycloaliphatic amines (IL-TMHMDA and IL-IPD), curing already started at room temperature after mixing the materials and the compositions could therefore not be stored for extended periods of time. The systems with the aromatic DDS hardener (IL-DDS) presented a higher latency. IL-DDS was stored in a fridge for several weeks and no pronounced phase separation or curing reaction was observed, which is in good agreement with the longer pot life and gel time of aromatic hardeners compared to aliphatic hardeners reported in literature [102,103]. The anhydride system (IL-MHHPA) also showed a starting curing reaction but can nonetheless be stored over extended periods (at least one week) of time if no accelerator is added. Typical DSC scans for the various epoxy-IL resins are depicted in Figure 2. Table 3 summarizes the characteristic temperatures of the uncured resin-hardener mixtures. The glass transition temperature, midpoint temperature in the second heating run of DSC tests, is denoted as *T*_g,u_. The results confirm the initial observation of the latency of IL-DDS having significantly higher temperatures of the exothermic peak (*T*_peak_) of 283 °C and curing onset (*T*_onset_) of 171 °C than the other epoxy-IL systems. A slightly smaller exothermic peak was observed at 230 °C. In consideration of the relatively low *T*_g,u_ of 65 °C for IL-DDS, the secondary exotherm was assigned to epoxy-IL homopolymerisation reactions. When comparing IL-DDS with DGEB-DDS, it can be seen that *T*_onset_ of 185 °C and *T*_peak_ of 238 °C for DGEB-DDS correspond very well with *T*_onset_ and the first exotherm of IL-DDS. This further indicates that the higher *T*_peak_ is derived from etherification [104]. It has to be noted that at such high temperatures some thermal degradation is likely to occur and a slight exotherm is visible when heating the pure IL-E to 300 °C in the DSC, however, no peak was visible. Epoxy-anhydride systems generally require a catalyst such as tertiary amines to initiate the alternating ring-opening polymerization and the onset temperature can be influenced by the variation of the catalyst concentration [105]. With 3 wt.% BDMA as catalyst, a curing onset of 120 °C and *T*_peak_ of 161 °C was measured for IL-MHHPA. Attempts to reduce the curing temperature for IL-DDS by addition of 1 wt.% BDMA resulted in a significant reduction of *T*_onset_ and *T*_peak_ to 88 °C and 135 °C, respectively. However, using the accelerator also resulted in a drastically reduced *T*_g,u_ value of 38 °C, indicating that BDMA mainly initiated the homopolymerisation of the IL, and was therefore disregarded. This observation is in good agreement with reports on catalyzed DGEB-DDS systems [106].

IL-IPD also exhibited a double exotherm at 108 °C and 161 °C. In this case, the double exotherm can be attributed to the different reactivity of the aliphatic and cycloaliphatic amine groups of the non-symmetrical IPD hardener [107]. This is further evidenced when comparing IL-IPD with IL-MHHPA and IL-TMHDA, cured with cycloaliphatic and aliphatic hardeners, respectively. The higher *T*_peak_ is at the same temperature as for IL-MHHPA. On the other hand, the *T*_onset_ of 63 °C and the lower *T*_peak_ at 108 °C are very similar to IL-TMHMDA, with a *T*_onset_ of 58 °C and a T_peak_ at 109 °C. The use of the aliphatic hardener in IL-TMHMDA also resulted in the lowest *T*_g,u_ of 32 °C. Apart from DGEB-DDS with 200 °C, the anhydride system IL-MHHPA exhibited the highest *T*_g,u_ of 99 °C, being even higher than the epoxy-IL system including the DDS hardener with its high aromatic content. IL-IPD with its mixed structure exhibits a *T*_g,u_ of 63 °C, ranking it between IL-TMHMDA and IL-MHHPA.

#### DSC Measurements of Cured Samples

**Figure 3:**
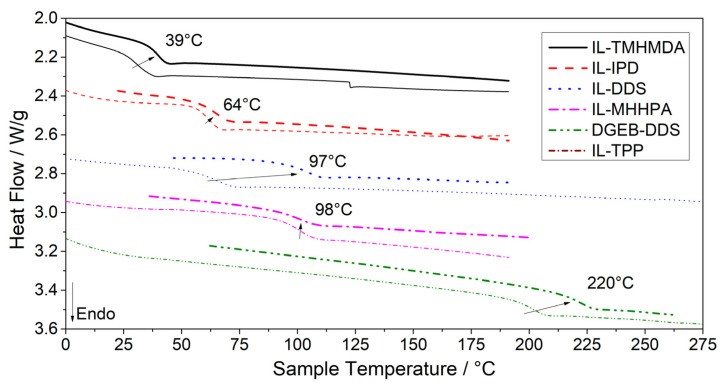
Shift of glass transition temperatures by prolonged post-curing (thick lines) compared to the second heating of uncured resins (thin lines) as determined in DSC. Curves shifted vertically for better visibility.

For a further investigation of the thermal properties, additional samples were prepared by resin casting and subsequent curing in a convection oven according to the determined temperature profiles for full curing. The curing temperatures were chosen in consideration of the respective *T*_onset_ and *T*_peak_ (see Table 3). Although the uncured resin samples did not exhibit an exothermic peak after the first heating run in DSC tests, a prolonged curing (post-curing) in the oven allowed to reach a higher glass transition temperature (*T*_g,c_) for the amine-cured systems. After reaching the gel-point, the curing reactions changes from kinetics-controlled to diffusion-controlled [108]. The additional curing time allows the diffusion-controlled curing process to proceed to a higher conversion, leading to a higher cross-link density and thus, higher *T*_g_. Figure 3 includes a comparison of the second heating curve from DSC tests with the compositions after post-curing to visualize the shift from *T*_g,u_ to *T*_g,c_. After post-curing, the glass transition temperature (*T*_g,c_) of IL-TMHMDA was increased by 8 °C to 39 °C and by 4 °C to 65 °C for IL-IPD, respectively. The discrepancy between *T*_g,u_ and *T*_g,c_ is especially pronounced for IL-DDS and a *T*_g,c_ of 97 °C compared to the *T*_g,u_ of 65 °C was reached. Interestingly, the IL-DDS system still exhibits a *T*_g_ of more than 100 °C lower compared to the respective DGEB-DDS system with a *T*_g,c_ of 220 °C. Compared to literature values of DGEBA cured with IPD with up to 155 °C [109], a similar relation can be observed for the *T*_g,c_ of IL-IPD at 64 °C. The anhydride cured system IL-MHHPA reached nearly 70% of reported values for DGEBA cured with MHHPA [110]. It is expected that this originates from the different molar and consequently weight ratios of the anhydride system. While the molar ratio of IL-E to DDS or IPD is 2:1, it is 1:1.7 for IL-MHHPA. Therefore, the final network properties are more significantly determined by the hardener structure for the anhydride system. Concluding from these results, the characteristic temperatures (*T*_g,c_, *T*_peak_ and *T*_g,u_) decrease in the order aromatic > cycloaliphatic > aliphatic for the amine curing agents, while the anhydride hardener provides a *T*_g_ comparable to the aromatic amine hardener (see Figure 3), which follows the same trend as conventional epoxy systems [110,111].

**Figure 4:**
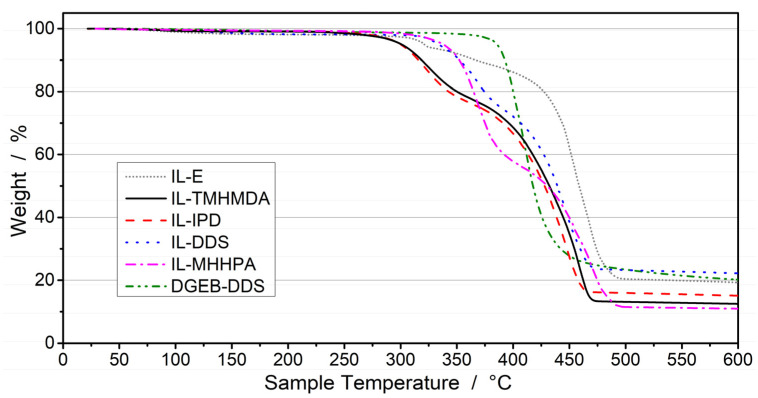
TGA curves obtained for IL-E and fully cured epoxy/hardener compositions.

TGA measurements (Figure 4) were done on fully cured epoxy-IL resins and DGEB-DDS systems in order to determine the thermal stability of the cured systems. The results are in good agreement with the high thermal stability of imidazolium based ionic liquids reported in literature [112]. The highest degradation temperature at 5% mass loss (*T*_dec,5_) was determined for DGEB-DDS at 385 °C. This high stability is explained by the highly aromatic content of the resin-hardener system. For the epoxy-IL samples, a two-step degradation is visible. The initial mass loss can be assigned to the degradation of the aliphatic moieties, initiated by the cleavage of the N-C bonds to the quaternized imidazolium nitrogen atoms. This is undermined by the significantly lower initial weight loss for the pristine, uncured IL-E and by the degradation of DGEB-DDS. The aromatic hardener DDS slightly increases the thermal stability of the cured IL-DDS network to a *T*_dec,5_ of 334 °C, above the stability of the pristine IL-E (*T*_dec,5_ = 321 °C). The cycloaliphatic hardener IPD, however, does not improve the thermal stability and is comparable to the linear aliphatic hardener TMHMDA, with *T*_dec,5_ of 300 °C and 301 °C for IL-IPD and IL-TMHMDA, respectively. A *T*_dec,5_ of 338 °C was measured for IL-MHHPA, indicating that the aliphatic amine of IPD might be the determining factor for the thermal stability.

### 3.2. Thermomechanical Properties of Thermally Cured IL-E

Dynamic mechanical analysis (DMA) was performed on cured specimens prepared by resin casting in order to characterize the temperature dependence of the mechanical properties and to estimate the stiffness of the materials. Storage modulus (*E*’) and glass transition temperature values as determined from these tests are shown in Table 4.

Curing of IL-TMHMDA led to comparably soft networks with a *T*_g_ of 28 °C and a storage modulus (*E*’) of 1.3 GPa. This behavior accounts for the lack of aromatic moieties between imidazolium groups, allowing for a higher chain mobility and decreasing the stiffness of the polymer backbone [113]. McDanel et al. [46] reported a similar *T*_g_ of 38 °C for the IL-E monomer cured with a triamine. However, the authors calculated a conversion of only 67%. An excess amount of hardener increased the conversion to 80% but at the same time reduced the *T*_g_ to 9 °C due to the introduction of further aliphatic groups. 

The implementation of the cycloaliphatic hardener in the IL-IPD resin system led to an increase of the *T*_g_ to 73 °C. Due to its high aromatic character, the IL-DDS system shows a further increase of the *T*_g_ to 113 °C. As discussed in the previous section, the comparably high *T*_g_ of 107 °C for the cured resin including the cycloaliphatic MHHPA hardener likely derives from the higher ratio of hardener to epoxy-IL resin. However, when compared to the DGEB-DDS system with a *T*_g_ of 220 °C, the glass transition of the epoxy-IL networks is still located at significantly lower temperatures. Apart from the different chemical structure of the heterocyclic imidazole, the increased volume due to the bulky anions implemented in the epoxy-IL networks and their plasticizing effect is expected to be the major influence on the decreased *T*_g_. While the Tf_2_N^−^ anion provides many desirable properties e.g., in terms of viscosity of the monomer and thermal stability, if further increase of the *T*_g_ is required, an anion exchange to reduce the free volume might be reasonable.

**Figure 5:**
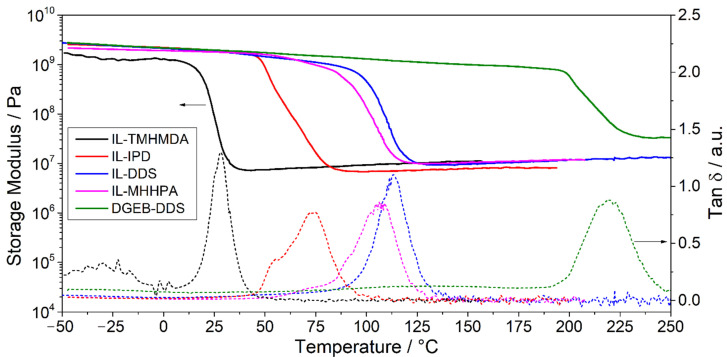
Storage modulus (solid lines) and tan δ (dashed lines) of the PIL networks and DGEB-DDS.

The non-symmetrical hardener IPD leads to a cured epoxy-IL network with a slightly broader glass transition and nearly 30 °C difference between the onset temperature and the peak of tan δ. A secondary relaxation temperature, visible as a shoulder in the tan δ curve (Figure 5), is observed for IL-IPD. This is accounted for by the chemical structure of IPD and the influence of the cycloaliphatic structure.

IL-IPD and IL-DDS exhibit storage moduli of 2.2 GPa and 2.1 GPa at 0 °C, respectively, similar to DGEB-DDS with 2.2 GPa, due to the rigid cyclic groups introduced by the hardeners. Interestingly, the anhydride cured network shows a lower E’ of 1.9 GPa at 0 °C, despite its high *T*_g_. At 31 °C, E’ of IL-MHHPA intersects with that of IL-DDS. However, E’ of IL-MHHPA increases less significantly with decreasing temperature. At −48 °C E’ amounts to 2.7 GPa and 2.6 GPa for IL-DDS and IL-IPD, respectively, while the modulus of IL-MHHPA only increased to 2.2 GPa. This indicates that the properties of the anhydride cured systems are less temperature dependent at low temperatures than the amine system, which might be advantageous for applications with large temperature differences.

Above the glass transition region, the epoxy-IL networks exhibit similar storage moduli in the rubbery state (*E*’_r_) of 7.0 MPa, 7.8 MPa, 9.8 MPa, and 10.0 MPa, following the order IL-IPD < IL-TMHMDA < IL-DDS < IL-MHHPA, while a *E*’_r_ of 33 MPa was measured for DGEB-DDS. This indicates that the IL-E determines the *E*’_r_ of the epoxy-IL networks and the effect of the hardener is less pronounced.

**Figure 6:**
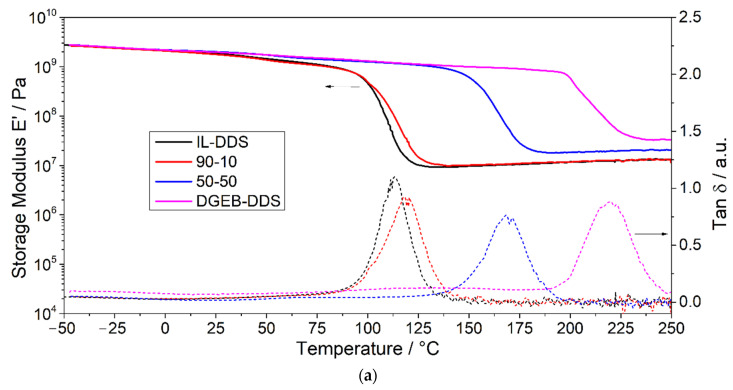

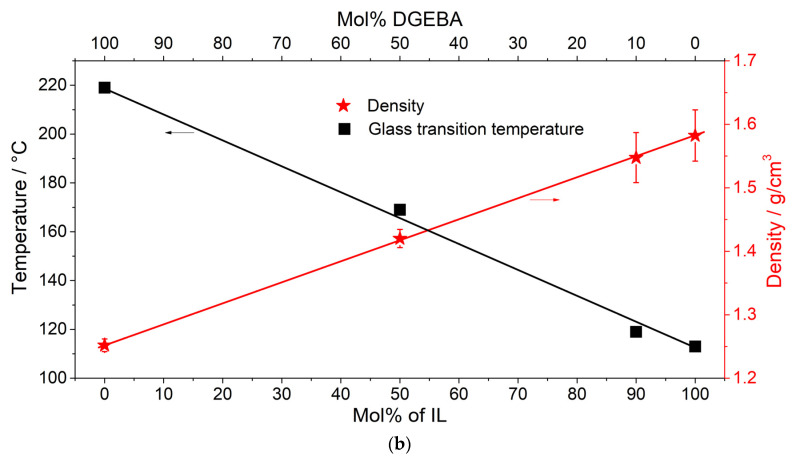
(**a**) Storage modulus (solid lines) and tan δ (dashed lines) of the copolymers. (**b**) *T*_g_ (■) and density (★) of the copolymers in dependence of the molar fractions of epoxy monomers.

Further networks were prepared by adding DGEBA as co-monomer and compositions with molar ratios of 1:0, 1:1, 9:1, and 0:1 of IL-E/DGEBA were produced and cured with DDS. The influence of the DGEBA content on the thermo-mechanical properties of the cured PIL networks was studied. The aim of these experiments was to determine the influence of the DGEBA content on the thermo-mechanical properties of the resulting networks, since conventional DGEBA resins have been extensively investigated in the past and DGEB-DDS networks provided good thermal stability and thermo-mechanical behavior in our experiments. Such blends of functionalized ionic liquids with other epoxy-based resins, which are not only limited to DGEBA, enable a high flexibility in tailoring the resulting network properties. In addition to the thermo-mechanical properties of the resulting epoxy-IL networks, also the ionic character and ion conductivity can therefore be flexibly tailored. Figure 6 depicts the DMA curves (a) as well as the glass transition temperatures and densities (b) of the networks in dependence of the molar fractions of epoxy monomers. Both the glass transition temperature and the density are observed to change linearly with the mol fractions. With increasing amounts of the IL monomer, the glass transition temperature of the thermoset decreases from 220 °C to 169 °C, 119 °C and 113 °C, while the density increases from 1.24 g/cm^3^ to 1.41 g/cm^3^, 1.58 g/cm^3^ and 1.63 g/cm^3^ for 50 mol%, 90 mol% and 100 mol% IL-E, respectively. This is in accordance with findings of other reports where imidazolium Tf_2_N derivatives were used as co-monomers [94,95]. The large Tf_2_N^−^ anion is reported to act as a plasticizer and leads to polymers with a low *T*_g_ [114]. The curing of DGEBA and epoxy-IL successfully yielded a homogenous network, as there are no additional relaxation temperatures visible and the glass transition range does not broaden. Therefore, while decreasing the ionic character of the material, the addition of DGEBA as co-monomer seems to be a suitable method to produce PILs for high thermomechanical demands. Compared to the copolyimides produced by Li et al. [73], the *T*_g_ is still significantly lower. However, the system is promising for lower *T*_g_ applications as the epoxy monomer is easier to process and does not require solvents, while higher IL loadings are achievable.

## 4. Conclusions

In the present work, the versatility of epoxy functionalized ionic liquids to achieve cross-linked poly(ionic liquids) with tunable properties was demonstrated. An epoxy-IL network was synthesized and the thermal and thermo-mechanical properties as a function of the applied hardener were evaluated. Due to the vast commercial use of conventional epoxy systems and the reactivity of the oxirane rings towards a multitude of species, cross-linked IL-thermosets can be produced easily, and the properties can be tailored by using commercially available hardeners. This was illustrated by adjusting the glass transition temperature of epoxy-IL networks from 30 °C to 100 °C using four different hardeners. The *T*_g_ was shifted to even higher temperatures, by adding DGEBA to the formulations. All reported networks only required thorough mixing and thermal curing, without the need of any solvent, for the preparation of free-standing specimens. This demonstrates the potential of epoxy-IL monomers for multiple technical applications and the possibilities of effectively tailoring the resulting resin properties by a suitable selection of hardener and co-monomer. Further evaluation of suitable alternatives to the implemented Tf_2_N^−^ anion opens up possibilities for cost effective epoxy-IL based monomers and possibly higher resulting *T*_g_ values, including the beneficial properties of ionic liquid monomers for certain applications.

## Figures and Tables

**Table 1:** Overview of PILs based on epoxy functional imidazolium ionic liquids reported in literature.

Monomer Structure	*T*_g_, °C	Ref.
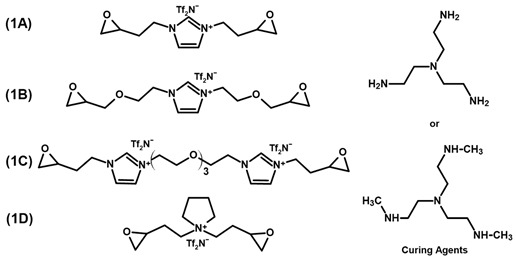	9/17/38 (A) −5/12 (B) −18/−27 (C) 3/20 (D)	[42,46]
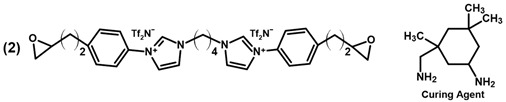	60 & 109	[34]
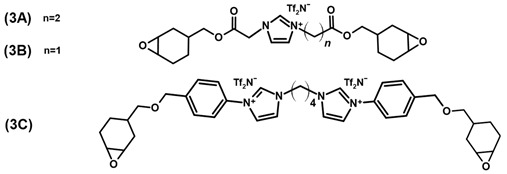	85 (3B)	[86]
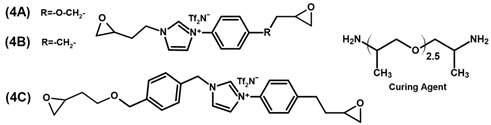	55 (A) 62 (B) 53 (C)	[36,49]
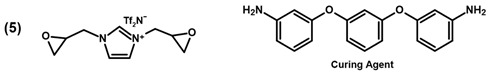	61	[79]

Tf_2_N^−^ = Bis(trifluoromethane)sulfonimide.

**Table 2:** Overview of the composition of the prepared epoxy-based resins and their respective cure cycles.

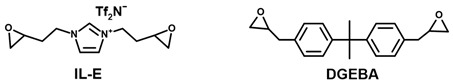			
Abbreviation	Epoxy Resin	Hardener	Cure Cycle
**IL-TMHMDA**	IL-E	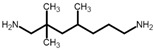 TMHMDA	60 °C (16 h) + 130 °C (1 h)
**IL-IPD**	IL-E	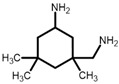 IPD	130 °C (16 h) + 160 °C (3 h)
**IL-MHHPA**	IL-E	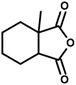 MHHPA	80 °C (1 h) + 140 °C (2 h) + 180 °C (30 min)
**IL-DDS**	IL-E	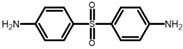 DDS	160 °C (16 h) + 180 °C (2 h)
**DGEB-DDS**	DGEBA		160 °C (16 h) + 180 °C (2 h)
**90-10**	90 mol% IL-E 10 mol% DGEBA		160 °C (16 h) + 180 °C (2 h)
**50-50**	50 mol% IL-E 50 mol% DGEBA		160 °C (16 h) + 180 °C (2 h)

**Table 3:** Characteristic temperatures obtained from DSC measurements performed on the uncured systems: *T*_onset_ and *T*_peak_ of the curing reaction (first heating), and glass transition temperature (*T*_g,u_) (second heating).

Resin/Hardener	*T*_onset_, °C	*T*_peak_, °C	*T*_g,u_, °C
**IL-TMHMDA**	58	109	32
**IL-IPD**	63	108 & 161	61
**IL-DDS**	171	230 & 283	65
**IL-MHHPA**	120	161	99
**DGEB-DDS**	177	241	200

**Table 4:** Glass transition temperatures and storage moduli obtained from DMA.

Material	*T*_g_, °C		*E*’, GPa *T* = 0 °C	*E*’_r_*, Mpa
	Onset	Max tan δ		
IL-TMHMDA	18	28	1.3	7.8
IL-IPD	44	73	2.2	7.0
IL-MHHPA	91	107	1.9	10.0
IL-DDS	99	113	2.1	9.8
DGEB-DDS	196	220	2.2	33

* *E*’_r_: Storage modulus in rubbery state (*T* = *T*(max tan δ) + 30 °C).

## Data Availability

The data presented in this study are available on request from the corresponding author.

## Supplementary Materials

The following are available online at https://www.mdpi.com/article/10.3390/polym13223914/s1, Figure S1: ^1^H-NMR spectrum of IL-E, Figure S2: 13C-NMR spectrum of IL-E, Figure S3: FTIR-spectrum of IL-E with corresponding characteristic frequency regions. References [46,97,98].

## References

[1] Zhao Q., Anderson J.L., Pawliszyn J. (2012). 2.11—Ionic Liquids. Comprehensive Sampling and Sample Preparation: Analytical Techniques for Scientists.

[2] Singh S.K., Savoy A.W. (2020). Ionic liquids synthesis and applications: An overview. J. Mol. Liq..

[3] Siriwardana A.I., Torriero A.A.J. (2015). Industrial Applications of Ionic Liquids. Electrochemistry in Ionic Liquids.

[4] Izgorodina E.I., Forsyth M., MacFarlane D.R. (2007). Towards a Better Understanding of ‘Delocalized Charge’ in Ionic Liquid Anions. Aust. J. Chem..

[5] Soares B.G., Livi S., Duchet-Rumeau J., Gerard J.-F. (2011). Synthesis and Characterization of Epoxy/MCDEA Networks Modified with Imidazolium-Based Ionic Liquids. Macromol. Mater. Eng..

[6] Styring P., Morreale B., Shi F. (2015). Chapter 7—Novel Sorbent Materials for Carbon Capture. Novel Materials for Carbon Dioxide Mitigation Technology.

[7] Harada L., Pereira J., Campos W., Silva E., Moutinho C., Vila M., Oliveira J., Teixeira J., Balcão V., Tubino M. (2018). Insights into Protein-Ionic Liquid Interactions Aiming at Macromolecule Delivery Systems. J. Braz. Chem. Soc..

[8] Walsh D.A., Goodwin S., Wandelt K. (2018). The Oxygen Reduction Reaction in Room-Temperature Ionic Liquids. Encyclopedia of Interfacial Chemistry: Surface Science and Electrochemistry.

[9] Marsh K.N., Boxall J.A., Lichtenthaler R. (2004). Room temperature ionic liquids and their mixtures—A review. Fluid Phase Equilibria.

[10] Vashchuk A., Fainleib A.M., Starostenko O., Grande D. (2018). Application of ionic liquids in thermosetting polymers: Epoxy and cyanate ester resins. Express Polym. Lett..

[11] Mai N.L., Ahn K., Koo Y.-M. (2014). Methods for recovery of ionic liquids—A review. Process. Biochem..

[12] Yusuf S.N.F., Yahya R., Arof A.K., Handy S. (2017). Ionic Liquid Enhancement of Polymer Electrolyte Conductivity and their Effects on the Performance of Electrochemical Devices. Progress and Developments in Ionic Liquids.

[13] Wang Q., Cai X., Liu Y., Xie J., Zhou Y., Wang J. (2016). Pd nanoparticles encapsulated into mesoporous ionic copolymer: Efficient and recyclable catalyst for the oxidation of benzyl alcohol with O_2_ balloon in water. Appl. Catal. B Environ..

[14] Hou W., Wang Q., Guo Z., Li J., Zhou Y., Wang J. (2017). Nanobelt α-CuV 2 O 6 with hydrophilic mesoporous poly(ionic liquid): A binary catalyst for synthesis of 2,5-diformylfuran from fructose. Catal. Sci. Technol..

[15] Zhang Z., Hu S., Song J., Li W., Yang G., Han B. (2009). Hydrogenation of CO_2_ to formic acid promoted by a diamine-functionalized ionic liquid. ChemSusChem.

[16] Kubisa P. (2004). Application of ionic liquids as solvents for polymerization processes. Prog. Polym. Sci..

[17] Nguyen T.K.L. (2019). New Generation of Epoxy Networks Based on Ionic Liquids: From Structuration to Final Properties. Ph.D. Thesis.

[18] Kumar V., Jamie Talisman I., Bukhari O., Razzaghy J., Malhotra S.V. (2011). Dual role of ionic liquids as phase transfer catalyst and solvent for glycosidation reactions. RSC Adv..

[19] Bugatti V., Viscusi G., Di Bartolomeo A., Iemmo L., Zampino D.C., Vittoria V., Gorrasi G. (2020). Ionic Liquid as Dispersing Agent of LDH-Carbon Nanotubes into a Biodegradable Vinyl Alcohol Polymer. Polymers.

[20] Booker K., Holdsworth C., Bowyer M., McCluskey A., Handy S. (2011). Ionic Liquids as Porogens in the Synthesis of Molecularly Imprinted Polymers. Applications of Ionic Liquids in Science and Technology.

[21] Bai L., Wang J., Zhang H., Liu S., Qin J., Liu H. (2015). Ionic liquid as porogen in the preparation of a polymer-based monolith for the separation of protein by high performance liquid chromatography. Anal. Methods.

[22] Zhang Y., Zhang S., Lu X., Zhou Q., Fan W., Zhang X. (2009). Dual amino-functionalised phosphonium ionic liquids for CO_2_ capture. Chemistry.

[23] Guerrero-Sanchez C., Ortiz-Alvarado A., Schubert U.S. (2009). Temperature effect on the magneto-rheological behavior of magnetite particles dispersed in an ionic liquid. J. Phys. Conf. Ser..

[24] Guerrero-Sanchez C., Wouters D., Hoeppener S., Hoogenboom R., Schubert U.S. (2011). Micellar dye shuttle between water and an ionic liquid. Soft Matter.

[25] Ribot J.C., Guerrero-Sanchez C., Greaves T.L., Kennedy D.F., Hoogenboom R., Schubert U.S. (2012). Amphiphilic oligoether-based ionic liquids as functional materials for thermoresponsive ion gels with tunable properties via aqueous gelation. Soft Matter.

[26] Ribot J.C., Guerrero-Sanchez C., Hoogenboom R., Schubert U.S. (2010). Thermoreversible ionogels with tunable properties via aqueous gelation of an amphiphilic quaternary ammonium oligoether-based ionic liquid. J. Mater. Chem..

[27] Shaplov A.S., Ponkratov D.O., Vlasov P.S., Lozinskaya E.I., Komarova L.I., Malyshkina I.A., Vidal F., Nguyen G.T.M., Armand M., Wandrey C. (2013). Synthesis and properties of polymeric analogs of ionic liquids. Polym. Sci. Ser. B.

[28] Tomé L.C., Isik M., Freire C.S.R., Mecerreyes D., Marrucho I.M. (2015). Novel pyrrolidinium-based polymeric ionic liquids with cyano counter-anions: High performance membrane materials for post-combustion CO_2_ separation. J. Membr. Sci..

[29] Li X., Zhang Z., Li S., Yang L., Hirano S.-i. (2016). Polymeric ionic liquid-plastic crystal composite electrolytes for lithium ion batteries. J. Power Sources.

[30] Pont A.-L., Marcilla R., de Meatza I., Grande H., Mecerreyes D. (2009). Pyrrolidinium-based polymeric ionic liquids as mechanically and electrochemically stable polymer electrolytes. J. Power Sources.

[31] Marcilla R., Blazquez J.A., Fernandez R., Grande H., Pomposo J.A., Mecerreyes D. (2005). Synthesis of Novel Polycations Using the Chemistry of Ionic Liquids. Macromol. Chem. Phys..

[32] Marcilla R., Alberto Blazquez J., Rodriguez J., Pomposo J.A., Mecerreyes D. (2004). Tuning the solubility of polymerized ionic liquids by simple anion-exchange reactions. J. Polym. Sci. Part A Polym. Chem..

[33] Tomé L.C., Aboudzadeh M.A., Rebelo L.P.N., Freire C.S.R., Mecerreyes D., Marrucho I.M. (2013). Polymeric ionic liquids with mixtures of counter-anions: A new straightforward strategy for designing pyrrolidinium-based CO_2_ separation membranes. J. Mater. Chem. A.

[34] Radchenko A.V., Chabane H., Demir B., Searles D.J., Duchet-Rumeau J., Gérard J.-F., Baudoux J., Livi S. (2020). New Epoxy Thermosets Derived from a Bisimidazolium Ionic Liquid Monomer: An Experimental and Modeling Investigation. ACS Sustain. Chem. Eng..

[35] Bhavsar R.S., Kumbharkar S.C., Rewar A.S., Kharul U.K. (2014). Polybenzimidazole based film forming polymeric ionic liquids: Synthesis and effects of cation–anion variation on their physical properties. Polym. Chem..

[36] Livi S., Lins L.C., Capeletti L.B., Chardin C., Halawani N., Baudoux J., Cardoso M.B. (2019). Antibacterial surface based on new epoxy-amine networks from ionic liquid monomers. Eur. Polym. J..

[37] Zhang S.-Y., Zhuang Q., Zhang M., Wang H., Gao Z., Sun J.-K., Yuan J. (2020). Poly(ionic liquid) composites. Chem. Soc. Rev..

[38] Hu X., Tang J., Blasig A., Shen Y., Radosz M. (2006). CO_2_ permeability, diffusivity and solubility in polyethylene glycol-grafted polyionic membranes and their CO_2_ selectivity relative to methane and nitrogen. J. Membr. Sci..

[39] Bhavsar R.S., Kumbharkar S.C., Kharul U.K. (2012). Polymeric ionic liquids (PILs): Effect of anion variation on their CO_2_ sorption. J. Membr. Sci..

[40] Kumbharkar S.C., Bhavsar R.S., Kharul U.K. (2014). Film forming polymeric ionic liquids (PILs) based on polybenzimidazoles for CO_2_ separation. RSC Adv..

[41] Bara J.E., Lessmann S., Gabriel C.J., Hatakeyama E.S., Noble R.D., Gin D.L. (2007). Synthesis and Performance of Polymerizable Room-Temperature Ionic Liquids as Gas Separation Membranes. Ind. Eng. Chem. Res..

[42] McDanel W.M., Cowan M.G., Barton J.A., Gin D.L., Noble R.D. (2015). Effect of Monomer Structure on Curing Behavior, CO_2_ Solubility, and Gas Permeability of Ionic Liquid-Based Epoxy–Amine Resins and Ion-Gels. Ind. Eng. Chem. Res..

[43] Bara J.E., Gabriel C.J., Hatakeyama E.S., Carlisle T.K., Lessmann S., Noble R.D., Gin D.L. (2008). Improving CO_2_ selectivity in polymerized room-temperature ionic liquid gas separation membranes through incorporation of polar substituents. J. Membr. Sci..

[44] Bara J.E., Hatakeyama E.S., Gin D.L., Noble R.D. (2008). Improving CO_2_ permeability in polymerized room-temperature ionic liquid gas separation membranes through the formation of a solid composite with a room-temperature ionic liquid. Polym. Adv. Technol..

[45] Carlisle T.K., Nicodemus G.D., Gin D.L., Noble R.D. (2012). CO_2_/light gas separation performance of cross-linked poly(vinylimidazolium) gel membranes as a function of ionic liquid loading and cross-linker content. J. Membr. Sci..

[46] McDanel W.M., Cowan M.G., Carlisle T.K., Swanson A.K., Noble R.D., Gin D.L. (2014). Cross-linked ionic resins and gels from epoxide-functionalized imidazolium ionic liquid monomers. Polymer.

[47] Leng Y., Wang J., Jiang P. (2012). Amino-containing cross-linked ionic copolymer-anchored heteropoly acid for solvent-free oxidation of benzyl alcohol with H_2_O_2_. Catal. Commun..

[48] Liu G., Hou M., Song J., Jiang T., Fan H., Zhang Z., Han B. (2010). Immobilization of Pdnanoparticles with functional ionic liquid grafted onto cross-linked polymer for solvent-free Heck reaction. Green Chem..

[49] Livi S., Chardin C., Lins L.C., Halawani N., Pruvost S., Duchet-Rumeau J., Gérard J.-F., Baudoux J. (2019). From Ionic Liquid Epoxy Monomer to Tunable Epoxy–Amine Network: Reaction Mechanism and Final Properties. ACS Sustain. Chem. Eng..

[50] Ohno H., Yoshizawa M., Ogihara W. (2004). Development of new class of ion conductive polymers based on ionic liquids. Electrochim. Acta.

[51] Green M.D., La Salas-de Cruz D., Ye Y., Layman J.M., Elabd Y.A., Winey K.I., Long T.E. (2011). Alkyl-Substituted N-Vinylimidazolium Polymerized Ionic Liquids: Thermal Properties and Ionic Conductivities. Macromol. Chem. Phys..

[52] Gu Y., Lodge T.P. (2011). Synthesis and Gas Separation Performance of Triblock Copolymer Ion Gels with a Polymerized Ionic Liquid Mid-Block. Macromolecules.

[53] Williams S.R., La Salas-de Cruz D., Winey K.I., Long T.E. (2010). Ionene segmented block copolymers containing imidazolium cations: Structure–property relationships as a function of hard segment content. Polymer.

[54] Dai Z., Ansaloni L., Gin D.L., Noble R.D., Deng L. (2017). Facile fabrication of CO_2_ separation membranes by cross-linking of poly(ethylene glycol) diglycidyl ether with a diamine and a polyamine-based ionic liquid. J. Membr. Sci..

[55] Kammakakam I., Bara J.E., Jackson E.M. (2020). Dual Anion–Cation Crosslinked Poly(ionic liquid) Composite Membranes for Enhanced CO_2_ Separation. ACS Appl. Polym. Mater..

[56] Matsumoto K., Endo T. (2010). Synthesis of networked polymers with lithium counter cations from a difunctional epoxide containing poly(ethylene glycol) and an epoxide monomer carrying a lithium sulfonate salt moiety. J. Polym. Sci. Part A Polym. Chem..

[57] Mecerreyes D. (2015). Applications of Ionic Liquids in Polymer Science and Technology.

[58] Shaplov A.S., Ponkratov D.O., Vlasov P.S., Lozinskaya E.I., Malyshkina I.A., Vidal F., Aubert P.-H., Armand M., Vygodskii Y.S. (2014). Solid-state electrolytes based on ionic network polymers. Polym. Sci. Ser. B.

[59] O’Harra K.E., Noll D.M., Kammakakam I., DeVriese E.M., Solis G., Jackson E.M., Bara J.E. (2020). Designing Imidazolium Poly(amide-amide) and Poly(amide-imide) Ionenes and Their Interactions with Mono- and Tris(imidazolium) Ionic Liquids. Polymers.

[60] Jin X.C., Guo L.Y., Deng L.L., Wu H. (2017). Study on epoxy resin modified by polyether ionic liquid. IOP Conf. Ser. Mater. Sci. Eng..

[61] Bernard F.L., Polesso B.B., Cobalchini F.W., Donato A.J., Seferin M., Ligabue R., Chaban V.V., do Nascimento J.F., Dalla Vecchia F., Einloft S. (2016). CO_2_ capture: Tuning cation-anion interaction in urethane based poly(ionic liquids). Polymer.

[62] Vlassi E., Pispas S. (2015). Imidazolium Quaternized Polymers Based On Poly(Chloromethyl Styrene) and their Complexes with FBS Proteins and DNA. Macromol. Chem. Phys..

[63] Dennis G.P., O’Harra K.E., Kammakakam I., Jones T.A., Mittenthal M.S., Flowers B.S., Tuan Y., Jackson E.M., Bara J.E. (2020). 6FDA -containing polyimide-ionene + ionic liquid gas separation membranes. J. Appl. Polym. Sci..

[64] Kammakakam I., O’Harra K.E., Dennis G.P., Jackson E.M., Bara J.E. (2019). Self-healing imidazolium-based ionene-polyamide membranes: An experimental study on physical and gas transport properties. Polym. Int..

[65] Bara J.E., O’Harra K.E., Durbin M.M., Dennis G.P., Jackson E.M., Thomas B., Odutola J.A. (2018). Synthesis and Characterization of Ionene-Polyamide Materials as Candidates for New Gas Separation Membranes. MRS Adv..

[66] Carlisle T.K., Bara J.E., Lafrate A.L., Gin D.L., Noble R.D. (2010). Main-chain imidazolium polymer membranes for CO_2_ separations: An initial study of a new ionic liquid-inspired platform. J. Membr. Sci..

[67] Hess M., Jones R.G., Kahovec J., Kitayama T., Kratochvíl P., Kubisa P., Mormann W., Stepto R.F.T., Tabak D., Vohlídal J. (2006). Terminology of polymers containing ionizable or ionic groups and of polymers containing ions (IUPAC Recommendations 2006). Pure Appl. Chem..

[68] Bara J.E., O’Harra K.E. (2019). Recent Advances in the Design of Ionenes: Toward Convergence with High-Performance Polymers. Macromol. Chem. Phys..

[69] O’Harra K.E., Kammakakam I., DeVriese E.M., Noll D.M., Bara J.E., Jackson E.M. (2019). Synthesis and Performance of 6FDA-Based Polyimide-Ionenes and Composites with Ionic Liquids as Gas Separation Membranes. Membranes.

[70] O’Harra K.E., Kammakakam I., Noll D.M., Turflinger E.M., Dennis G.P., Jackson E.M., Bara J.E. (2020). Synthesis and Performance of Aromatic Polyamide Ionenes as Gas Separation Membranes. Membranes.

[71] Hall L.M., Seitz M.E., Winey K.I., Opper K.L., Wagener K.B., Stevens M.J., Frischknecht A.L. (2012). Ionic aggregate structure in ionomer melts: Effect of molecular architecture on aggregates and the ionomer peak. J. Am. Chem. Soc..

[72] Evans C.M., Bridges C.R., Sanoja G.E., Bartels J., Segalman R.A. (2016). Role of Tethered Ion Placement on Polymerized Ionic Liquid Structure and Conductivity: Pendant versus Backbone Charge Placement. ACS Macro Lett..

[73] Li P., Coleman M.R. (2013). Synthesis of room temperature ionic liquids based random copolyimides for gas separation applications. Eur. Polym. J..

[74] Pascault J.-P., Williams R.J.J. (2010). Epoxy Polymers: New Materials and Innovations.

[75] Sinturel C., Thomas R., Thomas S. (2014). Micro- and Nanostructured Epoxy/Rubber Blends.

[76] White J.E., Brennan D.J., Silvis H.C., Mang M.N., Havelka K.O., McCormick C.L. (2000). Epoxy-Based Thermoplastics: New Polymers with Unusual Property Profiles. Specialty Monomers and Polymers.

[77] Parajó J.J., López M.V., Salgado J. Short-term thermal stability of ionic liquids. Proceedings of the 21st International Electronic Conference on Synthetic Organic Chemistry.

[78] Chardin C., Rouden J., Livi S., Baudoux J. (2017). Dimethyldioxirane (DMDO) as a valuable oxidant for the synthesis of polyfunctional aromatic imidazolium monomers bearing epoxides. Green Chem..

[79] Grugel R.N., Kaukler W.F., Paley M.S., Henry C.R., Canaday C.T., Hastings W.C., Rabenberg E. (2014). Development of Ionic Liquid Based Epoxies for Carbon Fiber Composite Cryogenic Tanks: National Space & Missile Materials Symposium (NSMMS), Huntsville. https://ntrs.nasa.gov/search.jsp?R=20140011777.

[80] Syakur A., Sutanto H. (2017). Determination of Hydrophobic Contact Angle of Epoxy Resin Compound Silicon Rubber and Silica. IOP Conf. Ser. Mater. Sci. Eng..

[81] Paley M.S., Libb R.S., Grugel R.N., Boothe R.E. (2008). Ionic Liquid Epoxy Resins. U.S. Patent.

[82] Demberelnyamba D., Yoon S.J., Lee H. (2004). New Epoxide Molten Salts: Key Intermediates for Designing Novel Ionic Liquids. Chem. Lett..

[83] Aslanov A.F., Korotkikh N.I., Shvaika O.P. (1996). Synthesis of 1,3-diglycidylimidazolium salts. Chem. Heterocycl. Compd..

[84] Lees I., Biyani K., Connor E., Hecker S., Zhang H., Cope M.J., Goka E., Lee A., Madsen D., Shao J. (2019). Polyimidazoles for Use as Bile Acid Sequestrants. U.S. Patent.

[85] Xu C., Yuan L., Liang G., Gu A. (2016). Building a poly(epoxy propylimidazolium ionic liquid)/graphene hybrid through π cation –π interaction for fabricating high-k polymer composites with low dielectric loss and percolation threshold. J. Mater. Chem. C.

[86] Radchenko A.V., Duchet-Rumeau J., Gérard J.-F., Baudoux J., Livi S. (2020). Cycloaliphatic epoxidized ionic liquids as new versatile monomers for the development of shape memory PIL networks by 3D printing. Polym. Chem..

[87] Rabenberg E., Brown A., Kaukler W.F., Grugel R.N. (2016). Evaluation of an ionic liquid-based epoxy after exposure on the MISSE-8 Carrier. Results Phys..

[88] Lyne C.T., Henry C.R., Kaukler W.F., Grugel R.N. (2018). Evaluation of ionic liquid epoxy carbon fiber composites in a cryogenic environment. Results Phys..

[89] Karr L., Curreri P., Paley M., Kaukler W., Marone M. (2012). Task-Specific Ionic Liquids for Mars Exploration (Green Chemistry for a Red Planet). LPI Contrib.

[90] Maksym P., Tarnacka M., Dzienia A., Matuszek K., Chrobok A., Kaminski K., Paluch M. (2017). Enhanced Polymerization Rate and Conductivity of Ionic Liquid-Based Epoxy Resin. Macromolecules.

[91] Perchacz M., Matějka L., Konefał R., Seixas L., Livi S., Baudoux J., Beneš H., Donato R.K. (2019). Self-Catalyzed Coupling between Brønsted-Acidic Imidazolium Salts and Epoxy-Based Materials: A Theoretical/Experimental Study. ACS Sustain. Chem. Eng..

[92] Yin J., Zhang C., Yu Y., Hao T., Wang H., Ding X., Meng J. (2020). Tuning the microstructure of crosslinked Poly(ionic liquid) membranes and gels via a multicomponent reaction for improved CO_2_ capture performance. J. Membr. Sci..

[93] Jiang Z., Wang Q., Liu L., Zhang Y., Du F., Pang A. (2020). Dual-Functionalized Imidazolium Ionic Liquids as Curing Agents for Epoxy Resins. Ind. Eng. Chem. Res..

[94] Zhang C., Cao B., Coleman M.R., Li P. (2016). Gas transport properties in (6FDA-RTIL)-(6FDA-MDA) block copolyimides. J. Appl. Polym. Sci..

[95] Li P., Zhao Q., Anderson J.L., Varanasi S., Coleman M.R. (2010). Synthesis of copolyimides based on room temperature ionic liquid diamines. J. Polym. Sci. Part A Polym. Chem..

[96] Demir B., Perli G., Chan K.-Y., Duchet-Rumeau J., Livi S. (2021). Molecular-Level Investigation of Cycloaliphatic Epoxidised Ionic Liquids as a New Generation of Monomers for Versatile Poly(Ionic Liquids). Polymers.

[97] Kiefer J., Fries J., Leipertz A. (2007). Experimental vibrational study of imidazolium-based ionic liquids: Raman and infrared spectra of 1-ethyl-3-methylimidazolium bis(trifluoromethylsulfonyl)imide and 1-ethyl-3-methylimidazolium ethylsulfate. Appl. Spectrosc..

[98] Boumediene M., Haddad B., Paolone A., Drai M., Villemin D., Rahmouni M., Bresson S., Abbas O. (2019). Synthesis, thermal stability, vibrational spectra and conformational studies of novel dicationic meta-xylyl linked bis-1-methylimidazolium ionic liquids. Journal of Molecular Structure.

[99] ISO/DIS6721-4 (2019). Plastics—Determination of Dynamic Mechanical Properties—Part 4: Tensile Vibration—Non-Resonance Method.

[100] (2014). ISO/DIS11357-2 Plastics—Differential Scanning Calorimetry (DSC)—Part 2: Determination of Glass Transition Temperature and Step Height.

[101] Burrell A.K., del Sesto R.E., Baker S.N., McCleskey T.M., Baker G.A. (2007). The large scale synthesis of pure imidazolium and pyrrolidinium ionic liquids. Green Chem..

[102] De Nograro F.F., Guerrero P., Corcuera M.A., Mondragon I. (1995). Effects of chemical structure of hardener on curing evolution and on the dynamic mechanical behavior of epoxy resins. J. Appl. Polym. Sci..

[103] Ozeren Ozgul E., Ozkul M.H. (2018). Effects of epoxy, hardener, and diluent types on the workability of epoxy mixtures. Constr. Build. Mater..

[104] Cole K.C., Hechler J.J., Noel D. (1991). A new approach to modeling the cure kinetics of epoxy/amine thermosetting resins. 2. Application to a typical system based on bis[4-(diglycidylamino)phenyl]methane and bis(4-aminophenyl) sulfone. Macromolecules.

[105] Anusic A., Resch-Fauster K., Mahendran A.R., Wuzella G. (2019). Anhydride Cured Bio-Based Epoxy Resin: Effect of Moisture on Thermal and Mechanical Properties. Macromol. Mater. Eng..

[106] Galy J., Sabra A., Pascault J.-P. (1986). Characterization of epoxy thermosetting systems by differential scanning calorimetry. Polym. Eng. Sci..

[107] Mounif E., Bellenger V., Mazabraud P., Nony F., Tcharkhtchi A. (2010). Chemorheological study of DGEBA/IPD system for reactive rotational molding (RRM). J. Appl. Polym. Sci..

[108] Su C.C., Woo E.M. (1997). Diffusion-controlled reaction mechanisms during cure in polycarbonate-modified epoxy networks. J. Polym. Sci. Part B Polym. Phys..

[109] Garcia F.G., Soares B.G., Pita V.J.R.R., Sánchez R., Rieumont J. (2007). Mechanical properties of epoxy networks based on DGEBA and aliphatic amines. J. Appl. Polym. Sci..

[110] Boey F.Y.C., Qiang W. (2000). Glass-transition temperature-conversion relationship for an epoxy-hexahydro-4-methylphthalic anhydride system. J. Appl. Polym. Sci..

[111] Ignatenko V.Y., Ilyin S.O., Kostyuk A.V., Bondarenko G.N., Antonov S.V. (2020). Acceleration of epoxy resin curing by using a combination of aliphatic and aromatic amines. Polym. Bull..

[112] Cao Y., Mu T. (2014). Comprehensive Investigation on the Thermal Stability of 66 Ionic Liquids by Thermogravimetric Analysis. Ind. Eng. Chem. Res..

[113] Krevelen D.W. (2009). Properties of Polymers: Their Correlation with Chemical Structure; Their Numerical Estimation and Prediction from Additive Group Contributions.

[114] Qian W., Texter J., Yan F. (2017). Frontiers in poly(ionic liquid)s: Syntheses and applications. Chem. Soc. Rev..

