# Supplementary material for: Cross-Linking and Evaluation of the Thermo-Mechanical Behavior of Epoxy Based Poly(ionic Liquid) Thermosets
Source: Polymers (Basel). 2021 Nov 12;13(22):3914. doi: 10.3390/polym13223914 (PMC8620924; doi:10.3390/polym13223914)
Supplement: Supplementary file 1 [file polymers-13-03914-s001.zip › polymers-1411669-SI.pdf]

Supplementary Materials

Cross-Linking and Evaluation of the Thermo-Mechanical Behavior of Epoxy Based Poly(Ionic Liquid) Thermosets

Florian Wanghofer, Archim Wolfberger *, Markus Wolfahrt and Sandra Schlögl

Polymer Competence Center Leoben GmbH, Roseggerstraße 12, 8700 Leoben, Austria; florian.wanghofer@pccl.at (F.W.); markus.wolfahrt@pccl.at (M.W.); sandra.schloegl@pccl.at (S.S.)

* Correspondence: archim.wolfberger@pccl.at

Spectroscopic data of 1,3-bis(2-oxiranyl-ethyl)imidazolium bis(trifluoromethane)sulfonimide (IL-E):

^{1}H-NMR (300 MHz, DMSO-d6): 1.90 (td, 2H), 2.16 (dd, 2H), 2.45 (d, 2H), 2.69 (t, 2H), 2.97 (d, 2H), 4.33 (t, 4H), 7.81 (s, 2H), 9.23 (s, 1H)

^{13}C-NMR (300 MHz, DMSO-d6): 32.21, 45.52, 46.38, 48.86, 122.59, 136.37

FTIR (ATR, cm^{-1}): 406, 509, 569, 599, 611, 653, 740, 763, 789, 841, 916, 1052, 1132, 1179, 1330, 1348, 1462, 1566, 3152

^{1}H-NMR (Figure S1) and FTIR (Figure S3) spectra confirm the chemical structure if IL-E and are in good agreement with data published by other authors [1]. Due to overlapping signals in the FTIR spectrum, not all signals could be individually assigned to the respective vibration modes. However, the obtained signals show good correlation with literature values [2,3]. Characteristic frequency ranges of the respective groups are additionally highlighted in Figure S3. In the ^{13}C-NMR spectrum, not all signals predicted for CF_3 groups of the anion in IL-E were detected. However, the anion's presence is confirmed in FTIR scans, and furthermore by the change of hydrophilicity to hydrophobicity during the metathesis reaction of bromide to bis(trifluoromethane)sulfonimide.

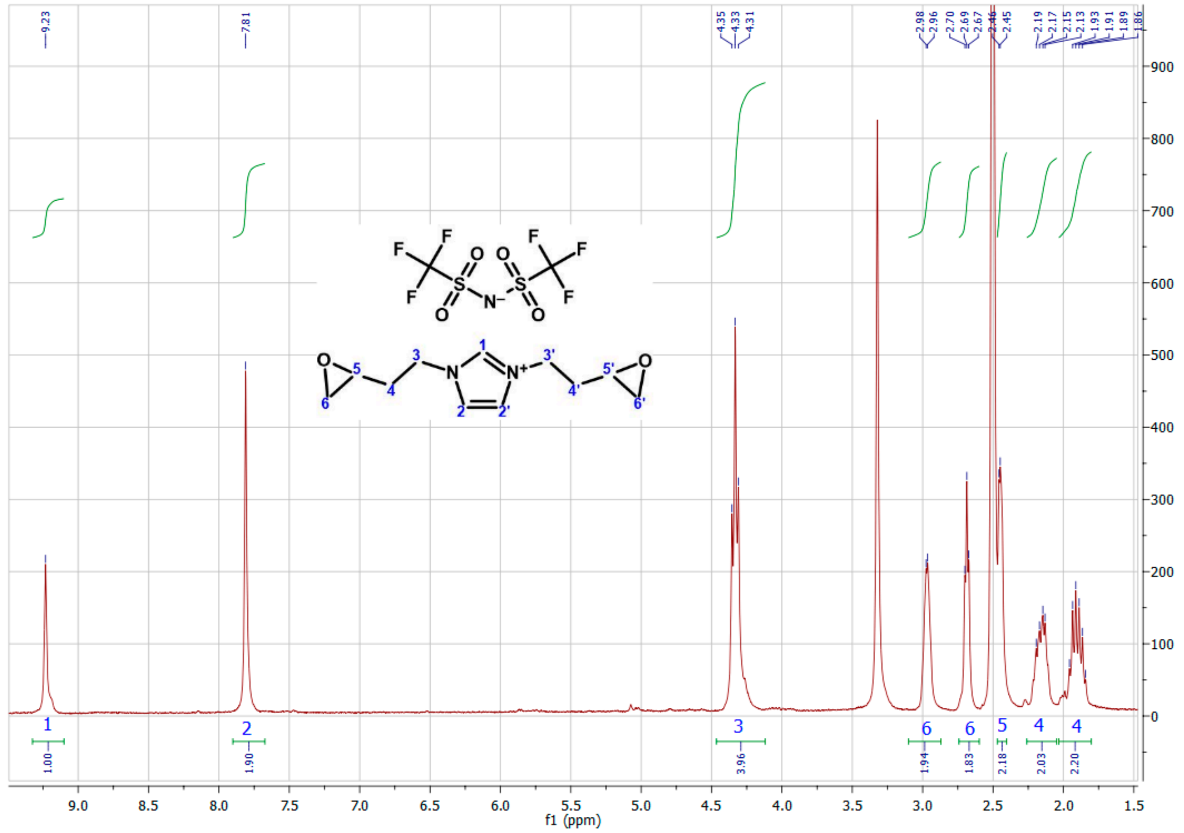

Figure S1. ^{1}H-NMR spectrum of IL-E.

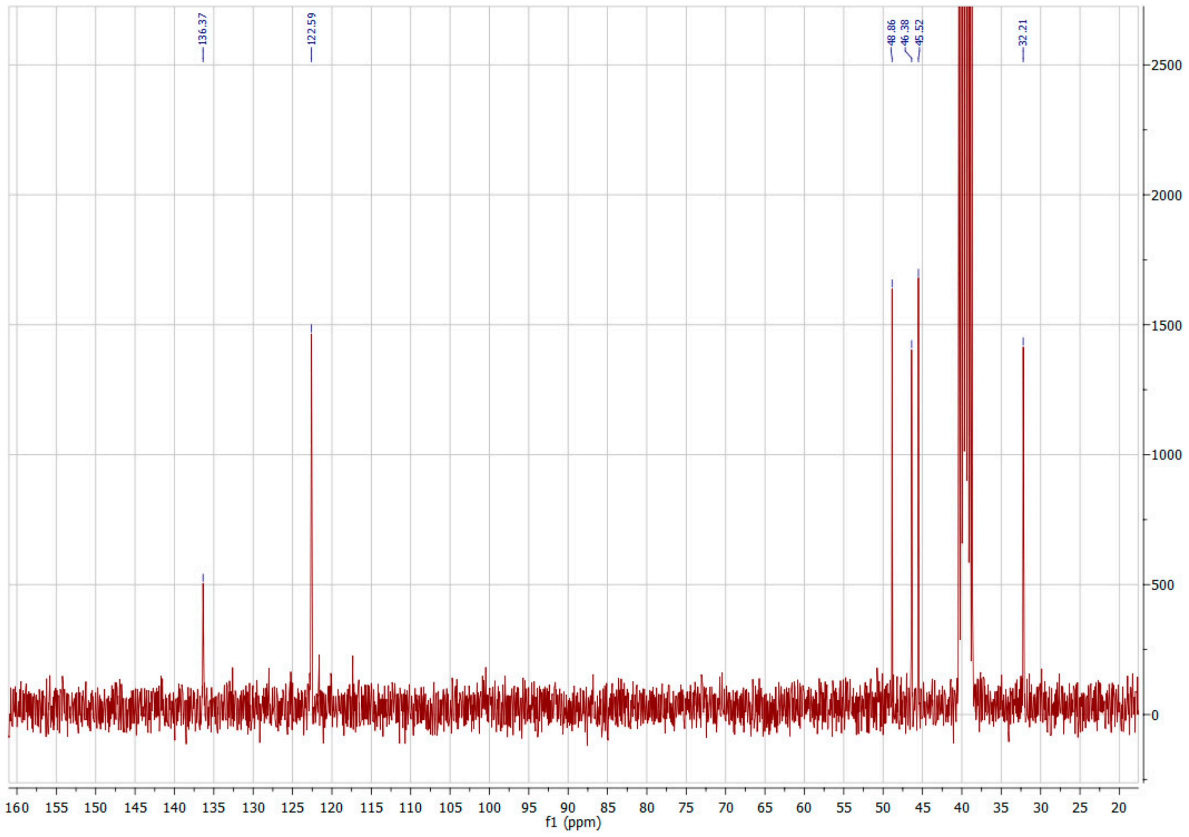

Figure S2. ^{13}C-NMR spectrum of IL-E.

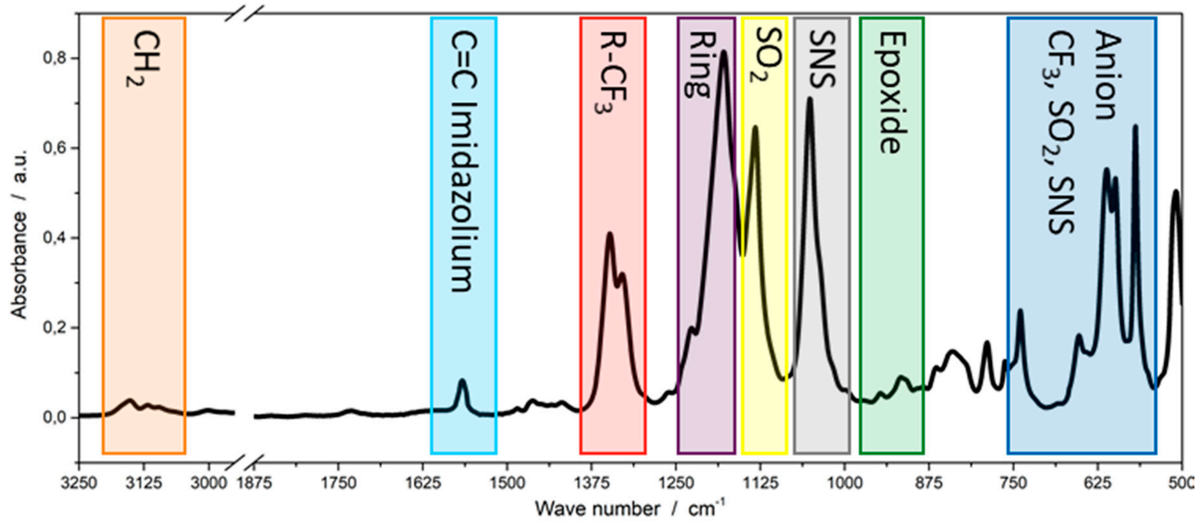

Figure S3. FTIR-spectrum of IL-E with corresponding characteristic frequency regions.

References

1. McDanel, W.M.; Cowan, M.G.; Carlisle, T.K.; Swanson, A.K.; Noble, R.D.; Gin, D.L. Cross-linked ionic resins and gels from epoxide-functionalized imidazolium ionic liquid monomers. *Polymer* **2014**, *55*, 3305–3313, doi:10.1016/j.polymer.2014.04.039.
2. Kiefer, J.; Fries, J.; Leipertz, A. Experimental vibrational study of imidazolium-based ionic liquids: Raman and infrared spectra of 1-ethyl-3-methylimidazolium bis(trifluoromethylsulfonyl)imide and 1-ethyl-3-methylimidazolium ethylsulfate. *Appl. Spectrosc.* **2007**, *61*, 1306–1311, doi:10.1366/000370207783292000.
3. Boumediene, M.; Haddad, B.; Paolone, A.; Drai, M.; Villemin, D.; Rahmouni, M.; Bresson, S.; Abbas, O. Synthesis, thermal stability, vibrational spectra and conformational studies of novel dicationic meta-xylyl linked bis-1-methylimidazolium ionic liquids. *Journal of Molecular Structure* **2019**, *1186*, 68–79, doi:10.1016/J.MOLSTRUC.2019.03.019.
